# Lifetime Prevalence and Correlates of Schizophrenia and Other Psychotic Disorders in Singapore

**DOI:** 10.3389/fpsyt.2021.650674

**Published:** 2021-03-11

**Authors:** Mythily Subramaniam, Edimansyah Abdin, Janhavi A. Vaingankar, Rajeswari Sambasivam, Yun Jue Zhang, Saleha Shafie, Sutapa Basu, Chun Ting Chan, Chuen Seng Tan, Swapna K. Verma, Charmaine Tang, Hong Choon Chua, Derrick Heng, Siow Ann Chong

**Affiliations:** ^1^Research Division, Institute of Mental Health, Singapore, Singapore; ^2^Saw Swee Hock School of Public Health, National University of Singapore, Singapore, Singapore; ^3^Department of Early Intervention in Psychiatry, Institute of Mental Health, Singapore, Singapore; ^4^Department of Psychosis and East Panel, Institute of Mental Health, Singapore, Singapore; ^5^CEO Office, Institute of Mental Health, Singapore, Singapore; ^6^Public Health Group, Ministry of Health, Singapore, Singapore

**Keywords:** psychotic disorders, prevalence, Singapore, multi-ethnic, survey

## Abstract

**Introduction:** The current study aimed to establish the lifetime prevalence of schizophrenia and other psychotic disorders, its sociodemographic correlates and association with physical disorders using data from the Singapore Mental Health Study (SMHS 2016).

**Methods:** A two-phase design comprising population-level screening of psychotic symptoms using the World Health Organization Composite International Diagnostic Interview version 3.0 psychosis screen followed by clinical reappraisal based on the Diagnostic and Statistical Manual of Mental Disorders, Fourth Edition (DSM-IV) criteria were used to establish the prevalence.

**Results:** A total of 6,126 respondents completed the first phase of the study, giving a response rate of 69.5%. 5.2% (*n* = 326) of respondents endorsed at least one symptom in the psychosis screen. After the phase two clinical reappraisal interviews and adjusting for false-negative rate, the corrected prevalence of schizophrenia and other psychotic disorders was 2.3% (95% CI: 2.3–2.3%). The odds of having DSM-IV schizophrenia and other psychotic disorders was significantly higher among those of Malay ethnicity (OR = 3.9, 95% CI 1.4–11.0), and those who were unemployed (OR = 4.3, 95% CI 1.2–15.9). 80.4% of those with a psychotic disorder had consulted a doctor or a mental health professional for their symptoms.

**Conclusions:** Our results indicate that approximately 2.3% of Singapore's community-dwelling adult population had a lifetime diagnosis of schizophrenia and other psychotic disorders. While the treatment gap of the disorder was relatively small, the severe nature of the disorder emphasizes the need for continued outreach and early diagnosis and treatment.

## Introduction

Schizophrenia and other psychotic disorders comprise a heterogeneous group of disorders including schizophrenia, schizophreniform disorder, schizoaffective disorder, delusional disorder, substance-induced psychotic disorder, brief psychotic disorder, and psychotic disorder not otherwise specified. These disorders are characterized by delusions, hallucinations, disorganized thoughts, and abnormal motor behavior (1). Lifetime prevalence estimates of schizophrenia and other psychotic disorders vary widely across population-based surveys, and this has been attributed to methodological differences (2) as well as genetic and environmental factors. Most epidemiological studies have used a single-phase design that relied on lay-administered fully structured interviews to derive a diagnosis of psychotic disorders which was not highly concordant with clinical reappraisal interviews (interviews conducted by trained clinicians to evaluate the agreement between lay interviewers' diagnoses and clinical diagnosis), conducted by psychiatrists (3, 4). The Hong Kong Mental Morbidity Survey (HKMMS) as well as the Health 2000 study in Finland using a two-phase design linking data from the survey with additional information (e.g., medical records), estimated the prevalence of any psychotic disorder to be 2.5 and 3.5%, respectively (4, 5). The China Mental Health Survey also used a two-phase design. Those screening positive in the psychosis screen and a random sample of those with negative screening results underwent a second phase assessment by psychiatrists who used the Structured Clinical Interview for the Diagnostic and Statistical Manual for Mental Disorders, Fourth Edition (DSM-IV) Axis I disorders (SCID) (Columbia University), and the study established the lifetime prevalence of schizophrenia and other psychotic disorders to be 0.7% (6). A recent systematic review and meta-analysis estimated that the pooled median point and 12-month prevalence of psychotic disorders was 3.9 and 4.0 per 1,000 persons, respectively, and the median lifetime prevalence was 7.5 per 1,000 persons (7).

While a relatively low prevalence disorder, schizophrenia and other psychotic disorders result in significant burden to the individual and society. People with schizophrenia and other psychotic disorders experience a higher risk of suicide (8, 9), substance abuse (10), and violence (11) as compared to the general population. Schizophrenia is also associated with poor physical health which further adds to the disease burden. Several studies have found a significant life expectancy gap in people with schizophrenia compared to the general population (12–14). The premature morbidity is largely contributed by deaths due to cardiovascular disease, cancer, and diabetes mellitus, which are often poorly diagnosed and not well-treated (15, 16). De Oliveira et al. (17) estimated the total direct cost of 142,821 patients over the age of 15 years (about 1.2% of the population) with chronic psychotic disorders in Ontario to be 2.1 billion CAD, in 2012 (approximately 1.6 billion USD). Neil et al. (18). estimated the average annual cost of psychotic disorders using data from the second Australian national survey of psychosis. The study established the 12-month treated prevalence of psychotic disorders in public treatment services to be 4.5 people per 1,000 (19). The average annual cost of psychotic disorders to society (in AUD) was $77,297 per affected individual, comprising $40,941 in lost productivity, $21,714 in health sector costs, and $14,642 in other sector costs annually. Psychosis was thus estimated to cost the Australian society $4.91 billion (approximately 2.9 billion USD) per annum.

Singapore is a multiracial country with a resident population of about 4 million comprising people belonging to the Chinese, Malay, Indian, and other ethnicities. Healthcare services in this city-state are provided through a mixed delivery model. Primary care is largely provided by private general practitioner (GP) clinics whereas the public sector dominates the hospitals, delivering 80% of acute care services. All the public and private hospitals in Singapore have mental health services that offer both inpatient and outpatient care. Primary care services provided by family care physicians and GPs also cater to those with psychological problems. Community based social services and voluntary welfare organizations (VWOs) provide counseling and support, rehabilitative services, and intermediate and long term care to those with mental health conditions. Knowing the extent of disease burden in the general population is important from the perspective of both service providers and policy makers for better development and distribution of health services as well as for the informed allocation of scarce resources. However, no study to date has examined the prevalence and correlates of schizophrenia and other psychotic disorders in Singapore. The aims of the current study were therefore to establish the prevalence of lifetime schizophrenia and other psychotic disorders (as defined by DSM-IV criteria) and to describe the profile in terms of socio-demographic factors, comorbid physical, and mental health conditions.

## Methods

### Survey Population and Subjects

The Singapore Mental Health Study initiated in 2016 (SMHS 2016) was a population-based, cross-sectional epidemiological study that included Singapore residents aged 18 years and above living in Singapore (20). The sampling frame was based on a national population registry database of all citizens and permanent residents in Singapore which is updated regularly. A probability sample was randomly selected using a disproportionate stratified sampling design with 16 strata defined according to ethnicity (Chinese, Malay, Indian, Others) and age groups (18–34, 35–49, 50–64, 65 and above). An over-sampling of residents aged 65 and above, and those of Malay and Indian ethnicity was done to ensure that sufficient sample size would be achieved to improve the reliability of estimates for the subgroup analysis. The study has been described in detail in an earlier article (21). The data were weighted to adjust for differential probability of selection of participants, non-response, and post-stratified by age and ethnicity between the survey sample and the Singapore resident population of 2014 ensuring that the survey findings were representative of the Singapore adult population.

The study was approved by the institutional ethics committee (National Healthcare Group, Domain Specific Review Board, Singapore) and all participants gave written informed consent before initiation of study procedures. Additionally, parental consent was sought for participants below 21 years of age as age of majority in Singapore is 21 years. Participants were paid SGD60 (approximately USD44) for their participation in Phase 1 and SGD50 (approximately USD37) for their participation in Phase 2 of the study. A resource brochure that contained helplines of the various mental healthcare providers including those in the hospitals, polyclinics, family service centers that provide counseling support, and other VWOs that provide mental health services, were given to all participants before initiation of the survey. Participants who met criteria for DSM-IV schizophrenia and other psychotic disorders during the clinical reappraisal interview, and not seeking treatment at the point of interview were given the resource brochure and also offered a consultation with a senior psychiatrist.

Six thousand one hundred and twenty-six respondents were interviewed in all which yielded a response rate of 69.5% and the acceptability of the interview was high as there were very few who agreed to begin the interview but did not complete it subsequently (incomplete cases = 25).

### Study Design

Taking reference from the HKMMS (4) and Australia's second national psychosis survey (19) a two-phase design was employed (22). This design is appropriate for estimating the prevalence of a relatively uncommon outcome and comprised a first-phase screening for psychotic symptoms followed by a second-phase of clinician-administered semi-structured interview to provide a more accurate estimation of lifetime prevalence of schizophrenia and other psychotic disorders.

### Questionnaires

#### WHO-CIDI

We used the fully-structured computer-assisted personal interview version of the World Health Organization Composite International Diagnostic Interview version 3.0 (WHO-CIDI 3.0) (23) to establish the prevalence of select mental disorders [major depressive disorder (MDD), bipolar disorder, generalized anxiety disorder (GAD), obsessive compulsive disorder (OCD), and alcohol use disorder (AUD) (which included alcohol abuse and dependence)] in the study. All diagnoses were made using organic exclusions and diagnostic hierarchy rules.

The psychosis screen which is a part of CIDI 3.0 was used to identify those likely to meet criteria for formal diagnosis of schizophrenia and other psychotic disorders.

The screen enquires about the lifetime occurrence of six symptoms (visual hallucinations, auditory hallucinations, thought insertion, thought control, delusions of reference, and delusions of persecution), with yes–no response options. Any positive response is followed by asking the respondent to confirm that these experiences occurred when they were not dreaming, not half asleep and not under the influence of alcohol or drugs (to ensure that hallucinations and delusions are in excess of those that typically accompany substance intoxication). Respondents who confirmed this, were then asked to describe the instances of the symptom and to provide what they believe to be the cause of the experience. These responses were recorded verbatim.

The verbatim records of the participants who endorsed one or more of the psychotic symptoms were reviewed by two of the researchers (MS and CSA) independently and assessed as “probable cases,” or “unlikely to meet criteria” for psychosis. When the two researchers disagreed on the rating (10 cases), the participant was assigned a probable rating to reduce the likelihood of missing a case (i.e., screening sensitivity was favored over screening specificity). One hundred and twenty-seven participants who were assessed to be probable cases of psychosis in the first phase were invited for the second phase of the study. An additional 127 participants were randomly selected from the pool of people who were screened negative for psychosis as “controls.” Their inclusion enabled estimation of prevalence without assuming that the psychosis screen had perfect sensitivity.

#### Clinical Reappraisal Interviews

**Figure 1:**
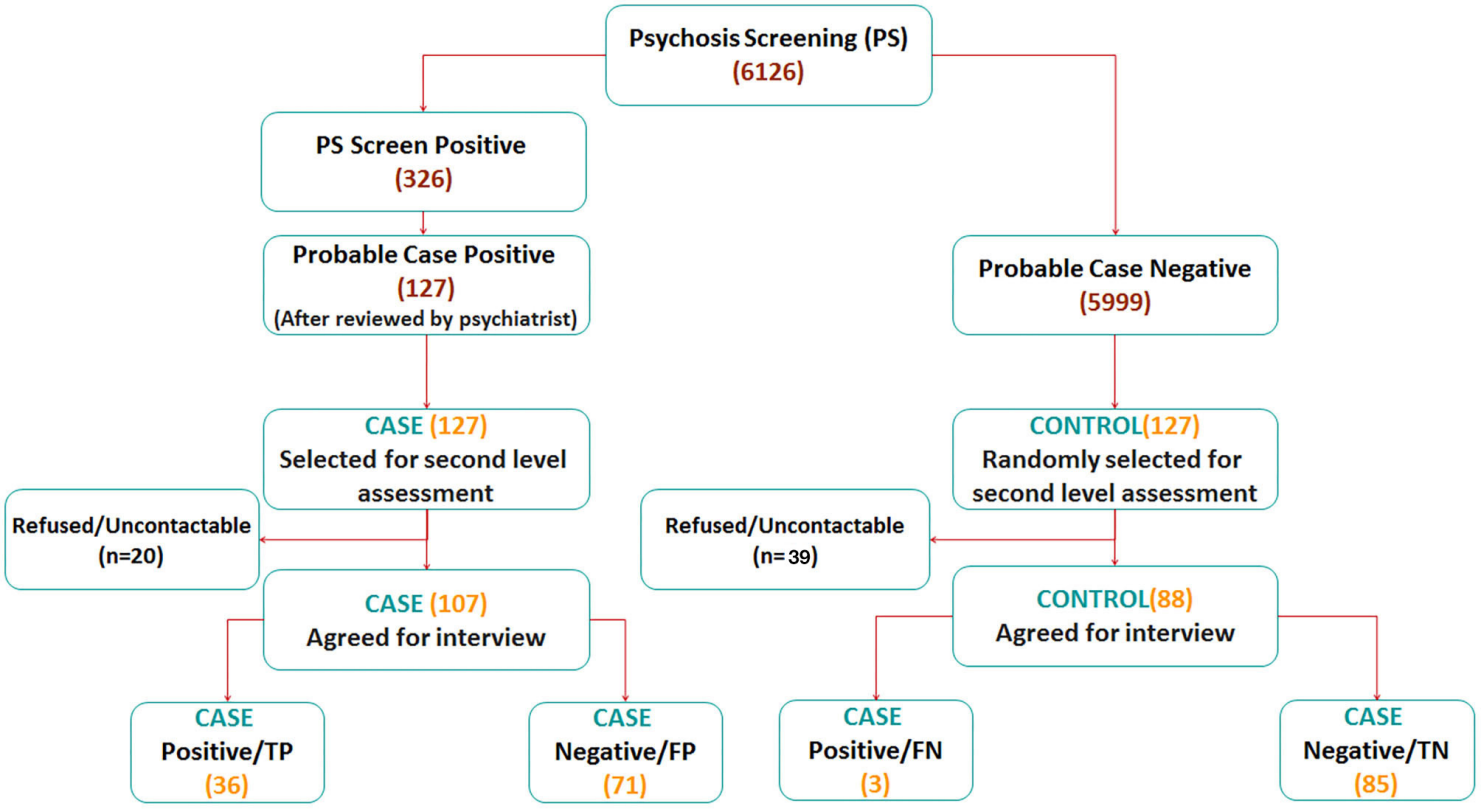
Participant flow diagram for phase 1 and phase 2 of the study.

These clinical assessments were conducted using a semi-structured clinical interview based on the SCID-I that included detailed information on the personal, medical, and psychiatric history of the respondent. Four experienced psychiatrists and one medically trained researcher (SAC, SV, SB, CCT, and MS) who were blind to the CIDI diagnosis of the respondents conducted the second phase, face-to-face interviews in the homes of the participant or any other preferred venue of their choice. The eventual diagnosis of schizophrenia and other psychotic disorders was made independently by each assessor using the DSM-IV checklist. In all, 195 interviews were completed in the second phase of the study (107 probable case and 88 control interviews) (Figure 1).

#### Assessment of Socio-Demographic and Other Correlates

Data on sex, age groups (18–34, 35–49, 50–64, and 65 years old and above), ethnicity (Chinese, Malay, Indian, and Others), marital status (single, married, divorced/separated or widowed), educational level (primary and below, secondary, vocational/technical education, pre-university/junior college, diploma, and university), employment status (employed, unemployed, and economically inactive i.e., students, homemakers, and retirees), and household income were collected. Information on chronic physical conditions as diagnosed by a doctor (self-report) were obtained; height and weight were taken using a tape measure and standardized weighing machine to calculate the body mass index (BMI). All the information was collected in the Phase 1 of the study and age, ethnicity, and educational level were reconfirmed in Phase 2 of the study.

### Statistical Analysis

To ensure that the survey findings were representative of the Singapore general population, the data were weighted and analyzed using survey analysis procedures. The final sample weights incorporated three different weights including sampling weights to adjust for oversampling by age and ethnicity, non-response adjustment weights, and post-stratification weights according to age and ethnicity of the Singapore adult population. Descriptive statistics were performed to describe the sociodemographic profile of the study population.

We estimated the prevalence of schizophrenia and other psychotic disorders using sampling weights derived from phase 1 and phase 2 data. Diagnostic status was ascertained at the clinical reappraisal interview and the proportion of screened people meeting criteria for a DSM-IV diagnosis of schizophrenia and psychotic disorders was estimated using appropriately weighted screen-positive and screen-negative sub-samples. A sensitivity analysis was conducted to take into account those who did not respond to the clinical appraisal. We assumed that all individuals who were positive for psychotic symptoms in the first phase and did not respond had either 50 or 100% increased risk of having schizophrenia and other psychotic disorders. A series of multivariable regression models were used to examine socio-demographic and lifestyle correlates of schizophrenia and other psychotic disorders. Association of schizophrenia and other psychotic disorders with BMI categories and chronic physical disorders was conducted using multinomial logistic and logistic regression analyses after adjusting for age, gender, and ethnicity. Statistical significance was set at the conventional level of *p* < 0.05, using two-sided tests.

## Results

### Sample Characteristics

The sample comprised 50.4% females and 49.6% males with a mean age of 45.2 years (range 18–97 years). 75.7% respondents were of Chinese descent, 12.5% were Malays, 8.7% were of Indian descent, and 3.1% belonged to other ethnic groups. The majority of the sample were married (59.8%) and employed (72.0%). We have also provided the Singapore population data for comparison. The adjusted data from our sample was largely similar to that of the population. Differences were noted in employment statistics as data from Singapore is limited to those aged 25–64 years, data for household incomes is also different as there were some methodological differences (Table 1).

**Table 1:** Socio-demographic distribution of the sample (*n* = 6,126).

**Socio-demographic characteristics**		***n***	**Unweighted %**	**Weighted %^#^**	Singapore Populatio*n* %^*^
Age group (years)	18–34	1,707	27.9	30.4	29.6
	35–49	1,496	24.4	29.6	29.7
	50–64	1,626	26.5	26.9	26.8
	65+	1,297	21.2	13.1	13.9
Gender	Women	3,058	49.9	50.4	50.9
	Men	3,068	50.1	49.6	49.1
Ethnicity	Chinese	1,782	29.1	75.7	74.3
	Malay	1,990	32.5	12.5	13.4
	Indian	1,844	30.1	8.7	9.1
	Others	510	8.3	3.1	3.2
Marital status	Never married	1,544	25.2	31.0	31.6
	Married	3,843	62.7	59.8	59.4
	Divorced/Separated	343	5.6	5.2	3.8
	Widowed	396	6.5	4.1	5.3
Education	Primary and below	1,187	19.4	16.3	
	Vocational Institute/Technical education	508	8.3	6.3	
	Below secondary				29.1
	Secondary	1,648	26.9	23.0	18.9
	Pre-U/Junior college	304	5.0	6.0	9.1
	Diploma	1,024	16.7	19.0	14.7
	University	1,455	23.8	29.4	28.2
Employment	Employed	4,055	66.2	72.0	80.3^##^
	Economically inactive^**^	1,716	28.0	22.7	
	Unemployed	354	5.8	5.3	4.1
Household income (SGD)^##^	No working person				10.8
	Below 2,000	1,147	21.0	16.5	7.5
	2,000–3,999	1,331	24.4	20.0	10.7
	4,000–5,999	1,113	20.4	21.4	11.3
	6,000–9,999	1,003	18.4	21.8	15.8
	10,000 and above	861	15.8	20.3	39.4

*SGD, Singapore Dollars*.

# *Weighted to adjust for oversampling by age and ethnicity, non-response adjustment weights, and post-stratification weights according to age and ethnicity of the Singapore adult population*.

## *Employment data is only for those aged 25–64 years*.

* *Singapore statistics from various Government websites for 2015–2016*.

** *Includes retirees, homemakers, and students*.

### Prevalence, Age of Onset, and Treatment Gap

The prevalence of psychotic symptoms based on endorsement of any of the six symptom questions from the CIDI 3.0 screener was 5.2% (*n* = 326). The most commonly endorsed symptoms in the population were visual hallucinations (3.3%), followed by auditory hallucinations (2.4%), persecutory delusions (0.3%), and telepathic powers (0.3%). The least commonly endorsed symptoms were thought insertion (0.2%) and thought control (0.1%).

Clinical review of the open-ended responses by two senior researchers with clinical experience (MS and CSA) concluded that 39% (*n* = 127) of respondents who endorsed these psychotic symptom questions (*n* = 326) were probable cases. Of these 127 cases, 107 agreed to a second interview of which 36 were assessed to meet criteria for a DSM-IV schizophrenia and other psychotic disorders (Figure 1).

The 199 cases that were excluded met one of the following criteria (i) experiencing a psychotic symptom just once in their lifetime, for example, those who reported experiencing a single episode of visual or auditory hallucination in their lifetime (ii) feeling the presence of a supernatural force in the local cultural context such as the Zhong Yuan Jie or Hungry Ghost Festival period (24). This festival celebrated by Taosist and Budhists falls on the seventh month of the lunar calendar (usually the month of August in the Western calendar). According to traditional beliefs, during this month, the gates of hell are opened and ghosts are released to wander on earth and look for food. During this month, those of Chinese ethnicity make offerings of joss sticks, candles, food, and paper money to the dead. Believers observe several customs during this period, which includes refraining from going out after dark, not making major changes to the house, not wearing red color, etc. They also avoid stepping on the offerings as one risks offending the hungry ghosts.

In all, 5,800 participants did not endorse any psychotic symptoms. After including those who were judged as not meeting criteria for probable case (*n* = 199); matched controls were chosen among 5,999 participants. Of these, only 88 agreed to be re-interviewed and three met criteria for schizophrenia and other psychotic disorders on the clinical reappraisal (Figure 1).

After adjusting for false negative rate in the clinical reappraisal interviews, the adjusted prevalence of schizophrenia and other psychotic disorders was 2.30% (95% CI: 2.30–2.30%). The adjusted prevalence of specific disorders was 0.86% (95% CI: 0.86–0.86%) for schizophrenia, 0.08% (95% CI: 0.08–0.08%) for schizoaffective, and 0.01% (95% CI: 0.01–0.01%) for delusional disorders. The prevalence of brief psychotic disorder was 0.01% (95% CI: 0.01–0.01%) and that of psychotic disorder not otherwise specified (Psychosis NOS) was 0.65% (95% CI: 0.65–0.65%) while prevalence of substance induced psychosis was 0.68% (0.68–0.68%) (Table 2).

**Table 2:** Lifetime prevalence of schizophrenia and other psychotic disorders in the SMHS 2016.

	**No. of participants**	**% (95% CI)^#^**
**Type of diagnosis**		
Schizophrenia	17	0.86 (0.86–0.86)
Schizoaffective disorder	4	0.08 (0.08–0.08)
Psychosis Not Otherwise Specified (NOS)	12	0.65 (0.65–0.65)
Substance induced psychosis	4	0.68 (0.68–0.68)
Delusional disorder	1	0.01 (0.01–0.01)
Brief psychotic disorder	1	0.01 (0.01–0.01)
**Schizophrenia, schizoaffective, and delusional disorder**	22	0.95 (0.95–0.95)
**Sensitivity analyses**		
All refusals at 0% increased risk	22	0.91 (0.90–0.91)
All refusals at 50% increased risk	32	1.07 (1.07–1.07)
All refusals at 100% increased risk	42	1.23 (1.23–1.24)
**Schizophrenia and other psychotic disorders (Total)**	39	2.30 (2.30–2.30)
**Sensitivity analyses**		
All refusals at 0% increased risk	39	2.20 (2.20–2.20)
All refusals at 50% increased risk	49	2.37 (2.37–2.37)
All refusals at 100% increased risk	59	2.50 (2.50–2.50)

# *Adjusted prevalence after adjusting for false negative rate*.

*Note: Due to rounding to two decimal places, some lower and upper limits of the 95% confidence intervals are displayed as equal*.

However, it must be noted that 18 respondents who had met criteria in the psychosis screen refused to undergo the second phase face-to-face interview and 2 respondents were uncontactable. A sensitivity analysis which assumed that all 20 cases had psychosis (100% increased risk), estimated the prevalence of schizophrenia and other psychotic disorders to be 2.5% (95% CI: 2.5–2.5%) while the prevalence of schizophrenia, schizoaffective, and delusional disorders was estimated to be 1.2% (95% CI: 1.2–1.2%). The mean age of onset of schizophrenia and other psychotic disorders was 23.1 years and 80.4% had consulted a doctor or a mental health professional for their symptoms.

### Sociodemographic Correlates

We found the odds of having lifetime schizophrenia and other psychotic disorders were significantly higher among Malays and those who were unemployed, and significantly lower among those who had higher household income (SGD 2,000–9,999 vs. <SGD 2,000) (Table 3).

**Table 3:** Sociodemographic correlates of lifetime schizophrenia and other psychotic disorders.

	**Schizophrenia and other psychotic disorders (*****n*** **=** **39)**			
	***n***	**%**	**OR (95% CI)**	***p*-value**
**Age group (years)**				
18–34	13	33.1	Ref	
35–49	13	43.3	1.8(0.4–8.0)	0.446
50–64	10	21.9	0.5(0.1–3.6)	0.499
65+	3	1.8	0.1(0.01–1.2)	0.066
**Gender**				
Female	21	52.4	Ref	
Male	18	47.6	0.8(0.2–2.7)	0.724
**Ethnicity**				
Chinese	4	46.1	Ref	
Malay	24	41.0	3.9(1.4–11.0)	0.001
Indian	7	8.7	1.3(0.3–4.8)	0.696
Others	4	4.2	1.9(0.4–9.6)	0.428
**Marital status**				
Married	16	35.6	Ref	
Never married	14	50.1	2.2(0.4–11.0)	0.348
Divorced/Separated	9	14.3	1.7(0.3–7.7)	0.524
Widowed	0	0	–	–
**Education**				
University	2	14.3	Ref	
Primary and below	11	25.7	2.8(0.5–15.0)	0.236
Secondary	13	31.2	1.8(0.4–7.7)	0.438
Pre-U/Junior college	2	1.3	0.5(0.1–4.2)	0.550
Vocational institute/Technical education	7	11.9	1.5(0.3–7.9)	0.560
Diploma	4	15.7	1.6(0.3–9.4)	0.574
**Employment**				
Employed	22	57.3	Ref	
Economically inactive	7	9.2	0.6(0.2–1.7)	0.324
Unemployed	10	33.5	4.3(1.2–15.9)	0.028
**Household income (SGD)**				
Below $2,000	22	63.6	Ref	
$2,000–$3,999	10	16.5	0.2(0.06–0.5)	0.002
$4,000–$5,999	1	2.0	0.03(0.003–0.2)	0.001
$6,000–$9,999	3	4.1	0.1(0.01–0.4)	0.003
$10,000 and above	2	13.8	0.3(0.05–1.9)	0.209

### Comorbidity With Physical Disorders

Majority of people (72.8%) with schizophrenia and other psychotic disorders had at least one chronic physical disorder. The most common comorbid chronic physical illness was chronic pain (29.4%), followed by hyperlipidemia (29.1%). After adjusting for age, gender, and ethnicity in a logistic regression model, schizophrenia, and other psychotic disorders were significantly associated with increased odds of having diabetes (OR 5.4). Prevalence of overweight and obesity was 37.8 and 22.5%, respectively, among those with schizophrenia and other psychotic disorders (Table 4).

**Table 4:** Association between lifetime schizophrenia and other psychotic disorders, body mass index categories and chronic physical disorders.

	**Schizophrenia and other psychotic disorders (*****n*** **=** **39)**				
	***n***	%^*^	**OR**	**95% CI**	***p*-value**
BMI classification^**^					
Obese	9	22.5	2.5	0.6–10.8	0.222
Overweight	13	37.8	2.4	0.8–7.5	0.135
Underweight	2	14.3	4.5	0.7–27.8	0.104
Normal	15	25.4	Ref.		
Chronic physical disorders^***^					
Hypertension	8	8.9	0.5	0.2–1.3	0.142
Hyperlipidemia	15	29.1	2.4	0.7–8.6	0.182
Diabetes	11	25.0	5.4	1.2–23.8	0.026
Asthma	12	17.0	1.3	0.6–3.0	0.507
Chronic pain	14	29.4	1.8	0.7–4.9	0.234
Cardiovascular disorders	2	1.4	0.5	0.1–1.9	0.278
Thyroid diseases	2	1.3	0.5	0.7–8.9	0.155
Ulcer	4	4.4	2.5	0.7–8.9	0.155
Cancer	0	·	·	·	·
Any chronic physical disorder	30	72.8	2.9	1.1–8.2	0.038

*OR, Odds Ratio*.

* *Prevalence of conditions among those with schizophrenia and other psychotic disorders*.

** *Multinomial logistic regression analysis adjusted for age, gender, and ethnicity*.

*** *Multiple logistic regression analysis adjusted for age, gender, and ethnicity*.

## Discussion

The overall weighted lifetime prevalence of any psychotic experience (PE) was 5.2% in the current study based on endorsement of any of the six symptom questions. Our rates are similar to that reported by McGrath et al. (25) using data from 18 countries as part of the World Health Organization World Mental Health Surveys. The authors found the mean lifetime prevalence of ever having a PE was 5.8. On the other hand, our prevalence figures were slightly lower than that reported by Linscott and van Os (26), who examined the prevalence of PE across 61 cohorts and found the median estimated prevalence of PEs to be 7.2%. The authors concluded that mode of interview (self-report vs. interview), and, urbanicity, significantly predicted between cohort variations.

This study is among the few that has established the prevalence of schizophrenia and other psychotic disorders using the DSM-IV criteria with a two-phase design (with clinical reappraisal). The study also concurrently examined the socio-demographic and clinical correlates of schizophrenia and other psychotic disorders. Our results showed that the lifetime prevalence of schizophrenia and other psychotic disorders was 2.3%. The prevalence was very similar to that reported in the HKMMS which employed a two-phase strategy (clinical interviews and medical record review in the second phase) and the two also share similarities in terms of being conducted in highly developed economies and among populations that are highly urbanized. The HKMMS found that the lifetime prevalence of non-affective psychosis (NAP) in the Hong Kong Chinese adult population was approximately 2.2% (5). The prevalence of NAP reported by the Finnish study was 1.9% which increased to 2.3% when they used register diagnosis for the non-responder group. However, this did not include substance induced psychotic disorder which was 0.4% in the population (4). Prevalence figures across all three studies were thus similar despite some differences in the methodology. This is in contrast to the China Mental Health Survey that found a relatively low prevalence of 0.7% of schizophrenia and other psychotic disorders with schizophrenia prevalence being 0.6% (6). The authors of the Chinese study suggested that the diverse nature of the psychotic symptoms as well as the stigma associated with the symptoms may have resulted in under-reporting in the face to face interview mode.

Prevalence of schizophrenia, schizoaffective, and delusional disorders was slightly lower at 0.9% in the current study in contrast to that of 1.3% in the HKMMS study (5), while that of psychosis NOS was higher in SMHS 2016 (0.6%) compared to HKMMS (0.38%). We suspect that some of those who met criteria for psychosis NOS would have met criteria for schizophrenia or schizoaffective disorder if the researchers had access to the medical records and other sources of information. It is possible that subjects who were clinically stable at the time of the interview were unable to remember the details of their illness or were unwilling to talk about their condition when they were most unwell. Self-reports of thought disorder and disorganization were difficult to elicit in the community setting. Given the ethical requirements of the study, the clinicians were not able to talk to family members and get any corroborative history. Thus, methodological limitations may have resulted in some misclassification of the disorder.

Our results were similar to others, and indicate that schizoaffective, brief psychotic, and delusional disorders are rare in the community setting (4, 5, 27). The prevalence of brief psychotic disorder may have been underestimated given the transient nature of the symptoms, and the prevalence of delusional disorder is also likely to be an underestimate, as those with this disorder often lack insight with relatively preserved functional capacity which could lead to both lack of treatment seeking and denial of symptoms.

The finding that psychotic disorders were associated with unemployment and low household income is in keeping with the literature, which indicates that individuals with the disorder are significantly more likely to be socioeconomically disadvantaged (5, 28). Given the cross-sectional nature of the study, it is difficult to determine if this was the result of social drift or social causation. While the social causation hypothesis asserts that experiencing economic hardship increases the risk of subsequent mental illness, it is also possible that the mental illness led to a fall in income and socioeconomic status (socioeconomic drift) (29). Other research has suggested that for schizophrenia, social selection (failure to ever attain high socio-economic status) plays a larger role with social drift (movement from higher to lower level jobs) having a more minor contribution (30, 31).

We did not find any gender differences in the prevalence of psychotic disorders. This is consistent with other epidemiological studies (4, 5, 32). Ochoa et al. (33) suggest that diagnostic criteria, medication compliance, and higher rates of suicides may result in the lack of gender differences observed in the prevalence while incidence may in fact be higher among men. Establishing the prevalence of schizophrenia and other psychotic disorders across different age-groups may provide a more nuanced and deeper understanding of gender differences but the low prevalence in the population sample limits our ability to conduct such analyses.

The association of Malay ethnicity with psychotic disorders is unique to this population as studies on multi-ethnic Asian populations are lacking. Razak (34) suggested that cultural and spiritual elements are significant in the perception of mental illness among Malays. Beliefs in genie possession and spirits, which are at times strengthened through religious teachings in this population, may lead to alternative illness explanatory models where psychosis may be viewed as a spiritual disturbance rather than a mental illness. A local study on illness perceptions among those with mental illness similarly found that those of Malay ethnicity were less likely to endorse biological causes of mental illnesses including schizophrenia (20). This may have led to a higher endorsement of symptoms and willingness to talk about the problem than among those belonging to other ethnic groups where such behaviors may be more stigmatized. Other studies have similarly found ethnic differences in the population but comparisons are difficult given that the results elsewhere are confounded by other geopolitical considerations such as immigration, homelessness, and rural-urban differences (35–37), which are not significant issues in Singapore. Examining the association between religiosity and PEs, data from 18 countries collected as part of the WHO world mental health surveys, revealed that there was no association between religious affiliations and PEs. However, among individuals who had a religious affiliation, four of five indices of religiosity (items that asked about the nature of their religious practice and/or spirituality) were significantly associated with increased odds of PEs (38). While our study did not have a measure of religiosity, it is possible that those of Malay ethnicity have increased religiosity which may be associated with increased odds of psychotic symptoms in the current sample.

Several studies have shown that the prevalence of diabetes among those with psychotic disorders exceeds that in the general population. The association between diabetes and psychosis is complex and multifactorial. In addition to risk factors like obesity, poor diet and sedentary lifestyle, other factors like the use of antipsychotic medication, shared genetic vulnerability and disparities in accessing healthcare among those with psychotic disorders may play a synergistic role (39). The comorbidity is concerning given that the treatment of comorbid diabetes and schizophrenia is fragmented across health care systems, and collaborative care models have been proposed to improve the medical care of patients with schizophrenia (40).

The treatment gap among those with schizophrenia and other psychotic disorders was low, with about 80% of them having sought help for their symptoms. This finding is similar to the HKMMS study where 79.8% of participants with any psychotic disorder had obtained some kind of professional help in the past year (5). However, studies elsewhere have identified a much larger treatment gap. Font et al. (41) found that 39.7% of those meeting criteria for psychotic disorders in France were not seeking help. A study from Ethiopia found that 41.8% among those identified with psychosis had not accessed biomedical care (42). Studies in Singapore have found that schizophrenia is poorly recognized, and stigmatized by the general public (43, 44). Surprisingly, the treatment gap of psychotic disorders is much lower than that of other mental disorders in Singapore (45). Singapore has a comprehensive network of mental health services—from VWOs in the community, to the Institute of Mental Health (IMH) a tertiary psychiatric hospital that offers a comprehensive range of services for people with severe mental illnesses. A specialized programme for early intervention in psychosis was initiated in 2001 which is supported by the Ministry of Health, Singapore and anchored in IMH. The Early Psychosis Intervention Programme (EPIP) works closely with healthcare professionals in other hospitals, polyclinics and social agencies to ensure early detection, referral, and management of psychotic disorders. EPIP also works with tertiary educational institutions for early identification and referral of cases (46). It also supports the Community Health Assessment Team (CHAT) which offers free mental health assessments for youth. CHAT is located in a youth friendly location within a shopping mall which reduces the stigma of help-seeking and increases the accessibility of mental health services for young people (47). The relatively low treatment gap in Singapore may be a result of the assertive outreach and networks established by the EPIP programme. It is also possible that in Singapore where the majority of the population lives in high-rise public housing, the close proximity of living with others also leads to early identification of those with psychosis whose manifestation would be generally more overt. Anecdotally these cases are highlighted by concerned neighbors to community workers and the local police who bring such cases to public hospitals for treatment.

## Limitations

Several limitations should be considered while interpreting the results of the study. First, the non-response of 30% to the overall survey as well as to the clinical reappraisal interview may have introduced bias in prevalence estimation. Nonetheless, the attrition effect was minimized by applying weighting procedure for non-response adjustment. Secondly, we did not include those who were incarcerated or institutionalized. As prevalence of psychosis in these populations is possibly higher than that in the community (48) our results are likely to be an underestimate of lifetime rates of psychotic disorders in the population. Thirdly, while we conducted clinical reappraisal interviews, we did not interview family members or access their medical records (of participants and/or non-participants) due to ethical reasons. This may have led to under-detection of cases. Fourthly, while the psychosis screen is sensitive, due to either stigma or social desirability bias participants may not have endorsed symptoms in the face to face interview with the lay interviewer. This led to identification of cases in the “control” group during the clinical reappraisal stage. While we have adjusted for this in our analysis it highlights under-reporting of psychotic symptoms in population studies. Fifth, there were significant differences in the sample population and that of Singapore in terms of household income, these may be due to methodological differences as well as over or under-reporting of income by the respondent. Lastly, the cross-sectional study design precluded us from drawing conclusions about any causal relationships.

## Conclusions

Our results indicate that approximately 2.3% of Singapore's adult population had a lifetime diagnosis of schizophrenia and other psychotic disorders. While the treatment gap of the disorder was relatively small, the severe nature of the disorder emphasizes the need for continued outreach and early diagnosis and treatment.

## Data Availability Statement

The raw data supporting the conclusions of this article will be made available by the authors, without undue reservation.

## Ethics Statement

The study involving human participants was reviewed and approved by National Healthcare Groups' Domain Specific Review Board. Written informed consent to participate in this study was provided by the participants' or their legal guardians/next of kin.

## Author Contributions

MS and EA wrote the first draft of the article. MS, EA, JV, DH, and SC designed the study protocol. SC, SV, CT, and HC designed the protocol for the second level assessments. EA and CST conducted the statistical analysis. RS, YZ, and SS conducted the literature review and provided logistic support for the survey and coordinated the second level assessments. MS, SB, CC, SV, and SC conducted the second level assessments. All the authors provided intellectual input into the final manuscript.

## Conflict of Interest

The authors declare that the research was conducted in the absence of any commercial or financial relationships that could be construed as a potential conflict of interest.

## Abbreviations

AUD: Australian Dollar
AUD: Alcohol Use Disorder
CAD: Canadian Dollar
CHAT: Community Health Assessment Team
CI: Confidence Interval
DSM-IV: Diagnostic and Statistical Manual for Mental Disorders, Fourth Edition
EPIP: Early Psychosis Intervention Programme
GAD: Generalized Anxiety Disorder
GP: General Practitioner
HKMMS: Hong Kong Mental Morbidity Survey
IMH: Institute of Mental Health
MDD: Major Depressive Disorder
NAP: Non-Affective Psychosis
OCD: Obsessive-Compulsive Disorder
OR: Odds Ratio
PE: Psychotic Experiences
Psychosis NOS: Psychotic Disorder Not Otherwise Specified
SCID: Structured Clinical Interview for the Diagnostic and Statistical Manual for Mental Disorders, Fourth Edition Axis I disorders
SGD: Singapore Dollar
SMHS 2016: Singapore Mental Health Study 2016
USD: United States Dollar
VWO: Voluntary Welfare Organizations
WHO-CIDI: World Health Organization Composite International Diagnostic Interview version 3.0.
